# Ticks on wild vertebrates necropsied in a veterinary pathology service in central Brazil: species richness and pathogen screening

**DOI:** 10.1590/S1984-29612026001

**Published:** 2026-05-15

**Authors:** Igor Silva Silito, Matheus Pasini-Martins, Isabella Pereira Pesenato, Marcelo Bahia Labruna, Márcio Botelho de Castro, Liz de Albuquerque Cerqueira, Thiago Fernandes Martins

**Affiliations:** 1 Universidade de São Paulo - USP, Faculdade de Medicina Veterinária e Zootecnia - FMVZ, Departamento de Medicina Veterinária Preventiva e Saúde Animal - VPS, Laboratório de Doenças Parasitárias - LDP, São Paulo, SP, Brasil; 2 Universidade Estadual de Campinas - UNICAMP, Instituto de Biologia, Departamento de Biologia Animal, Divisão de Parasitologia, Campinas, SP, Brasil; 3 Universidade de Brasília - UnB, Faculdade de Agronomia e Medicina Veterinária, Laboratório de Patologia e Medicina Veterinária Forense, Brasília, DF, Brasil

**Keywords:** Ixodidae, Amblyomma, wildlife, Rickettsia, Allocryptoplasma, Federal District, Ixodidae, carrapatos, vida silvestre, Rickettsia, Allocryptoplasma, Distrito Federal

## Abstract

During 2022-2024, fresh carcasses of 35 wild animals from Goiás state and the Federal District, both in central Brazil, were sent to the veterinary pathology service of the University of Brasília. The carcasses were examined for the presence of ticks, which were collected and sent to the laboratory for taxonomic identification. A total of 541 tick specimens were collected from the following host species: birds – *Aramides cajaneus* and *Cariama cristata*; mammals – *Coendou prehensilis*, *Didelphis albiventris*, *Hydrochoerus hydrochaeris*, *Myrmecophaga tridactyla*, *Sapajus libidinosus*, *Subulo gouazoubira* and *Tapirus terrestris.* Nine tick species were identified: *Amblyomma brasiliense*, *Amblyomma dubitatum*, *Amblyomma longirostre*, *Amblyomma nodosum*, *Amblyomma ovale*, *Amblyomma sculptum*, *Amblyomma triste, Ixodes loricatus*, *Rhipicephalus microplus*. We report here, for the first time, nymphs of *A. dubitatum* on *A. cajaneus* and *T. terrestris*; as well as the first record of *A. brasiliense* in the Central-Western region of Brazil. Molecular analyses revealed the presence of the human pathogen *Rickettsia parkeri* strain Atlantic rainforest in *A. ovale,* and the following agents of unknown pathogenicity: *Rickettsia amblyommatis* in *A. longirostre; Rickettsia bellii* in *A. longirostre, A. nodosum* and *I. loricatus*; *Rickettsia* sp. strain COOPERI in *A. nodosum,* and ‘*Candidatus* Allocryptoplasma sp.’ in *A. nodosum.*

## Resumo

Durante 2022-2024, carcaças frescas de 35 animais selvagens do estado de Goiás e do Distrito Federal foram enviadas ao serviço de patologia veterinária da Universidade de Brasília. As carcaças foram examinadas quanto à presença de carrapatos, que foram recolhidos e enviados ao laboratório para identificação taxonômica. Um total de 541 espécimes de carrapatos foram coletados das seguintes espécies hospedeiras: aves – *Aramides cajaneus* e *Cariama cristata*; mamíferos – *Coendou prehensilis, Didelphis albiventris, Hydrochoerus hydrochaeris, Myrmecophaga tridactyla, Sapajus libidinosus, Subulo gouazoubira* e *Tapirus terrestris*. Foram identificadas nove espécies de carrapatos: *Amblyomma brasiliense, Amblyomma dubitatum, Amblyomma longirostre, Amblyomma nodosum, Amblyomma ovale, Amblyomma sculptum, Amblyomma triste, Ixodes loricatus* e *Rhipicephalus microplus*. Relatamos aqui, pela primeira vez, ninfas de *A. dubitatum* em *A. cajaneus* e *T. terrestris*, bem como o primeiro registo de *A. brasiliense* na região Centro-Oeste do Brasil. Análises moleculares revelaram a presença do patógeno humano *Rickettsia parkeri* cepa Mata Atlântica em *A. ovale*, e dos seguintes agentes de patogenicidade desconhecida: *Rickettsia amblyommatis* em *A. longirostre*; *Rickettsia bellii* em *A. longirostre, A. nodosum* e *I. loricatus*; *Rickettsia* sp. cepa COOPERI em *A. nodosum* e ‘*Candidatus* Allocryptoplasma sp.’ em *A. nodosum*.

## Introduction

Conservation Medicine, a concept that has been widely disseminated since the 1990s, aims to understand the complex interactions between health, ecology, and human activities, investigating how anthropogenic changes, such as habitat degradation and fragmentation, forest fires, urban expansion, road construction, and increased air and water pollution impact ecosystems, compromise wildlife health, and consequently increase the risk of transmission of pathogens and emerging diseases to human populations (Karesh & Cook, 1995). Most emerging infectious diseases in humans originate in non-human animals, and human-induced environmental changes are the driving forces behind the emergence of new pathogens, often resulting from zoonotic spillover (Ellwanger et al., 2022). In scenarios of habitat destruction and land-use changes, increased contact between wild animals and their ticks occurs, as ticks, obligate bloodsuckers that feed on a wide range of vertebrates, including humans, are considered the second most important vectors of human diseases and the primary vectors of pathogens in domestic and wild animals (Yu et al., 2015).

Among the nearly 80 species of ticks of the Brazilian fauna, the vast majority use wild vertebrates as hosts. Ticks are known vectors of zoonotic pathogens, among which *Rickettsia rickettsii* stands out as the causative agent of the tick-borne zoonosis Brazilian Spotted Fever (BSF) (Oliveira et al., 2016). Another tick-borne rickettsiosis affecting humans is caused by *Rickettsia parkeri* sensu stricto and *R. parkeri* strain Atlantic rainforest, both of which have already been reported in Brazil (Labruna, 2009; Krawczak et al., 2016; Faccini-Martínez et al., 2018; Nieri-Bastos et al., 2018). Other zoonotic pathogens - such as bacteria of the genera *Anaplasma, Ehrlichia, Borrelia* and *Coxiella,* and protozoa of the order Piroplasmida - also rely on wild animals in their enzootic cycles, with ticks as their main vectors. Ticks, along with mosquitoes, are considered the most important arthropod vectors of infectious diseases (Sonenshine & Roe, 2013).

In addition to the risk of pathogen transmission through direct contact with injured animals, it is also important to highlight the exposure risk faced by professionals in veterinary pathology laboratories, especially those working in anatomical pathology diagnostics, as a potentially vulnerable group. Thus, the aim of this study was to evaluate which tick species parasitize wild animals in Goiás state and the Federal District, and to investigate the possible infection of these ticks by pathogens of relevance to One Health, within a Diagnostic Pathology Service located in Brazil’s Central-Western region.

## Material and Methods

### Study area and sample collection

**Figure 1:**
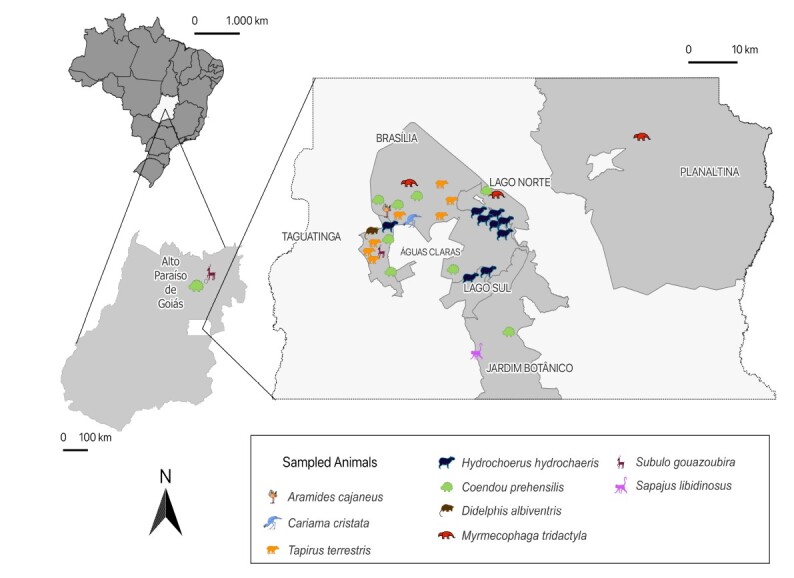
Municipality in Goiás and administrative regions of the Federal District where wild animals were captured.

Between 2022 and 2024, ticks were collected from wild animals necropsied by the Diagnostic Pathology Service of the Laboratory of Veterinary Pathology and Forensics at the Veterinary Hospital of the University of Brasília (LPPV-HVET-UNB). All animals lived freely in the wild and had been referred by CETAS-DF (Wildlife Screening and Rehabilitation Center of the Federal District) and ICMBio (Chico Mendes Institute for Biodiversity Conservation), some as roadkill victims. They originated from the municipality of Alto Paraíso de Goiás, GO (14° 8' 2.346" S, 47° 30' 50.378" W), and from administrative regions of the Federal District: Brasília (15° 47' 39.224" S, 47° 52' 55.798" W), Planaltina (15° 37' 17.85" S, 47° 39' 7.744" W), and Santa Maria (16° 0' 13.014" S, 47° 59' 14.168" W). Geographic coordinates of wild animal collection sites were recorded via Global Positioning System and mapped using QGIS (Figure 1).

All collected ticks were stored in 1.5 ml conical tubes with 70% ethanol and sent to the Laboratory of Parasitic Diseases of the Department of Preventive Veterinary Medicine and Animal Health, School of Veterinary Medicine and Animal Science, University of São Paulo, for identification and further processing.

### Tick identification

Tick samples were identified to the species and life stage level using a stereomicroscope and taxonomic identification keys (Martins et al., 2024). Subsequently, part of the tick samples was deposited in the tick collection "Coleção Nacional de Carrapatos Danilo Gonçalves Saraiva” (CNC), while the remaining samples were frozen at -80°C until processing.

### Molecular analyses

After taxonomic identification, DNA was extracted from a subset of the tick specimens using the guanidine isothiocyanate method (Sangioni et al., 2005), followed by a conventional PCR targeting the mitochondrial 16S rRNA gene found in ectoparasites to validate the DNA extraction and to prevent false negatives (Mangold et al., 1998).

The extracted DNA was screened for *Rickettsia* DNA using a TaqMan real-time PCR assay (7500 Real Time PCR Systems – Applied Biosystems, Foster City, CA, USA) with primers CS-5 (Guedes et al., 2005) and CS-6 (Labruna et al., 2004), targeting a 147 bp fragment of the *Rickettsia gltA* gene, including an internal fluorogenic probe, as described (Labruna et al., 2004). Samples testing positive by real-time PCR were further analyzed using conventional PCR with primers CS2 CS-78F and CS2 CS-323R, targeting a 401 bp fragment of the *gltA* gene (Labruna et al., 2004). Additionally, *gltA*-positive samples were tested by a second PCR assay using primers Rr190.70 and Rr190.701, which amplify a 632 bp fragment of the *ompA* gene, characteristic of *Rickettsia* species within the Spotted Fever Group (SFG) (Roux et al., 1996). Tick samples that tested negative with the *ompA* protocol were tested using a species-specific PCR targeting the *gltA* gene of the non-SFG species *Rickettsia bellii*, as previously described (Szabó et al., 2013). This latter assay was used to avoid sequencing all samples that tested positive in the first *gltA* PCR but negative for *ompA*. All PCR runs included a positive control (*Rickettsia vini* DNA cultured in Vero cells) and a negative control (ultrapure water type I). For the species-specific PCR for *R. bellii*, DNA cultured in Vero cells from the *R. bellii* Mogi strain was used as a positive control.

Tick DNA samples were also tested by a two additional TaqMan real-time PCR assays (each one targeting the bacterial genus *Borrelia* or *Coxiella*), and several conventional PCR assays targeting members of the bacterial family Anaplasmataceae (e.g., genera *Anaplasma* and *Ehrlichia*), and protozoa of the order Piroplasmida (genera *Babesia*, *Cytauxzoon* and *Theileria*), as listed in Table 1. For the PCR protocol targeting the family Anaplasmataceae, *Ehrlichia canis* DNA was used as a positive control; for *Borrelia* spp., DNA from *Borrelia anserina* was used. In the protocol for *Coxiella* spp., DNA from *Coxiella burnetii* strain At12 cultured in Vero cells was used, and for Piroplasmida, DNA from *Babesia bigemina* served as the positive control. In all reactions, ultrapure water type I was used as the negative control.

**Table 1:** PCR primers and probes used in the present study for the detection of tick-borne organisms.

**Gene/Primers**	**Targeted organisms**	**Primer sequence (5´- 3´)** ^a^	**Amplicon size**	**Reference**
**16S rRNA**	**Anaplasmataceae**			
EHR16SF		F-GGTACCYACAGAAGAAGTCC	344 pb	Inokuma et al. (2000)
EHR16SR		R-TAGCACTCATCGTTTACAG		
**16S rRNA**	***Borrelia* spp.**			
Bor16S3F		F-AGC CTT TAA AGC TTC GCT TGT AG^b^	148 pb	Parola et al. (2011)
Bor16S3R		R-GCC TCC CGT AGG AGT CTG G^b^		
Probe		6FAM (CCG GCC TGA GAG GGT GGT GAA CGG^b^		
***icd***	***Coxiella* spp.**			
COX-CbF		F-TCC ATC GTG ACT TAC CAA CAC ATC^b^	246 pb	Fernández-Carrillo et al. (2023)
COX-CbR		R-GCC ATA CAC TTT CGT AGC C^b^		
**18S rRNA**	**Piroplasmida**			
BAB2 143–167		F-CCG TGC TAA TTG TAG GGC TAA TAC A	551 pb	Almeida et al. (2012)
BAB2 694-667		R-GCT TGA AAC ACT CTA RTT TTC TCA AAG		
***gltA***	***Rickettsia* spp.**			
CS2 CS-78		F-GCA AGT ATC GGT GAG GAT GTA AT	401 pb	Labruna et al. (2004)
CS2 CS-323		R-GCT TCC TTA AAA TTC AAT AAA TCA GGAT		
CS-5		F-GAGAGAAAATTATATCCAAATGTTGAT^b^	147 pb	Guedes et al. (2005)
CS-6		RAGGGTCTTCGTGCATTTCTT^b^		Labruna et al. (2004)
Probe		6-FAM d(CATTGTGCCATCCAGCCTACGGT) BHQ-1^b^		Labruna et al. (2004)
***ompA***	***Rickettsia* spp. SFG**			
190.70		F-ATG GCG AAT ATT TCT CCA AAA	632 pb	Roux et al. (1996)
190.701		R-GTT CCG TTA ATG GCA GCA TCT		
***gltA***	***Rickettsia bellii***			
RbelliiF		5´-ATC CTG ATT TGC TGA ATT TTT T-3´	338 pb	Szabó et al. (2013)
RbelliiR		5´-TGC AAT ACC AGT ACT GAC G-3´		

a F: Foward, R: Reverse;

b used in a Taqman real-time PCR assay.

Amplified products were analyzed by electrophoresis on 1.5% agarose gels stained with SYBR Safe DNA Gel Stain (Life Technologies, Grand Island, NY, USA) and visualized under UV transillumination. All PCR products of the expected size were purified with ExoSap (USB, Cleveland, OH, USA) and sequenced (bidirectional Sanger sequencing) on an automated ABI sequencer (Applied Biosystems/Thermo Fisher Scientific, ABI 3500 Genetic Analyzer, Foster City, CA, USA) using the same primers as in the PCR. The resulting sequences were compared to one another and submitted to BLAST analysis (www.ncbi.nlm.nih.gov/blast) to determine the closest matches available in GenBank.

## Results

A total of 35 free-living wild animals (from at least nine different species and eight vertebrate orders) were necropsied: 2 birds: *Aramides cajaneus* (Müller, 1776) and *Cariama cristata* (Linnaeus, 1766), in addition to 33 mammals of the following species: *Coendou prehensilis* (Linnaeus, 1758), *Hydrochoerus hydrochaeris* (Linnaeus, 1766), *Didelphis albiventris* Lund, 1840, *Myrmecophaga tridactyla* Linnaeus, 1758, *Sapajus libidinosus* (Spix, 1823), *Subulo gouazoubira* (Fischer, 1814), and *Tapirus terrestris* (Linnaeus, 1758). All animals were parasitized by ticks (Ixodida: Ixodidae), which were collected, identified, and listed in Table 2, according to host species, locality, year of collection, tick species and life stages.

**Table 2:** Origin of the wild animals sampled in the present study, and the ticks (L: larva; N: nymph; M: male; F: female) collected from them.

**Hosts**	**Locality**	**Municipality/administrative region**	**Date**	**Tick species: n. per stage**	**CNC** **^2^** **deposit number**
**ORDER CARIAMIFORMES**					
*Cariama cristata*	Cetas-DF^1^	Brasília - DF	20/09/2022	*Amblyomma sculptum* 16 N	4836
**ORDER GRUIFORMES**					
*Aramides cajaneus*	Cetas-DF^1^	Brasília - DF	27/07/2023	*A. sculptum* 3 N; *Amblyomma dubitatum* 1 N	4835
**ORDER DIDELPHIMORPHIA**					
*Didelphis albiventis*	Cetas-DF^1^	Brasília - DF	18/05/2023	*Ixodes loricatus*^+^ 1 M	*
*D. albiventis*	Cetas-DF^1^	Brasília - DF	10/04/2023	*A. sculptum* 1 F	4837
**ORDER PILOSA**					
*Myrmecophaga tridactyla*	Lago Norte	Brasília - DF	11/09/2022	*A. sculptum* 4 N; *Amblyomma nodosum*^+^ 1 F, 1 M	4841
*M. tridactyla*	Cetas-DF^1^	Planaltina - DF	01/03/2022	*A. nodosum* 1 M	*
*M. tridactyla*	Cetas-DF^1^	Brasília - DF	12/09/2023	*A. sculptum* 1 F, 77 N; *A. nodosum* 5 M	4841
**ORDER PERISSODACTYLA**					
*Tapirus terrestris*	Brasília National Park	Brasília - DF	04/07/2022	*A. sculptum* 4 F	4843
*T. terrestris*	ICMBio^1^	Brasília - DF	22/08/2022	*Amblyomma brasiliense* 1 F, 1 M, 1 N; *A. sculptum* 1 F, 3 M, 46 N	4843
*T. terrestris*	ICMBio^1^	Brasília - DF	02/06/2023	*A. sculptum* 23 F, 35 M, 24N; *Amblyomma dubitatum*^+^ 1 N; *Amblyomma* sp. 2 L	4843
*T. terrestris*	Cetas-DF^1^	Brasília - DF	27/07/2023	*A. sculptum* 3 F, 1 M	4843
*T. terrestris*	Granja do Torto	Brasília - DF	13/04/2024	*A. sculptum* 2 F, 6 M; *Amblyomma triste* 1F	4843
*T. terrestris*	Brasília	Brasília - DF	04/06/2024	*A. sculptum* 45 F, 22 M, 34 N; *A. brasiliense* 2 M; *A. triste* 1 M; *Amblyomma* sp. 3 L	4975
*T. terrestris*	Brasília National Park	Brasília - DF	20/09/2024	*A. sculptum* 1N	4976
**ORDER ARTIODACTYLA**					
*Subulo gouazoubira*	ICMBio^1^	Brasília - DF	11/07/2022	*A. sculptum* 1 N; *Amblyomma ovale*^+^ 1 F; *Rhipicephalus microplus* 9 F, 1 M	4839
*S. gouazoubira*	Chapada dos Veadeiros	Alto Paraíso - GO	2/6/2022	*R. microplus* 4 F, 4 M, 1 L	4840
**ORDER RODENTIA**					
*Hydrochoerus hydrochaeris*	Lago Norte	Brasília - DF	12/08/2022	*A. sculptum* 20 M, 6 N; *A. dubitatum* 1 M	4842
*H. hydrochaeris*	ICMBio^1^	Brasília - DF	11/04/2023	*A. sculptum* 3 F; *A. dubitatum* 1 M	4842
*H. hydrochaeris*	DIVAL^1^	Brasília - DF	09/07/2024	*A. dubitatum* 1 M, 1 N	4842
*H. hydrochaeris*	Paranoá Lake	Brasília - DF	26/08/2024	*A. sculptum* 1 N; *A. dubitatum* 1 M, 14 N	4842
*H. hydrochaeris*	Lago Norte	Brasília - DF	09/08/2024	*A. sculptum* 1 N; *A. dubitatum* 3 F	4842
*H. hydrochaeris*	Lago Norte	Brasília - DF	22/08/2024	*A. sculptum* 1 M	4842
*H. hydrochaeris*	Parque das Garças, Lago Norte	Brasília - DF	21/09/2024	*A. sculptum* 4 M, 1 F, 1 N; *A. dubitatum* 6 M, 1 N	4977
*H. hydrochaeris*	Lago Sul	Brasília - DF	07/11/2024	*A. sculptum* 8 M, 6 F; *A. dubitatum* 3 M	4978
*H. hydrochaeris*	Lago Sul	Brasília - DF	12/12/2024	*A. sculptum* 13 M, 14 F; *A. dubitatum* 4 M, 1 F, 1 N	4978
*Coendou prehensilis*	Cetas-DF^1^	Brasília - DF	13/09/2022	*A. sculptum* 1 M, 1 N; *Amblyomma longirostre* 1 M	4838
*C. prehensilis*	Cetas-DF^1^	Brasília - DF	05/10/2022	*A. sculptum* 3 N; *A. longirostre*^+^ 3 F, 3 M	4838
*C. prehensilis*	Chapada dos Veadeiros	Alto Paraíso - GO	20/06/2023	*A. longirostre*+ 2 M	*
*C. prehensilis*	Cetas-DF^1^	Brasília - DF	27/07/2023	*A. longirostre* 4 M	4838
*C. prehensilis*	Cetas-DF^1^	Brasília - DF	02/08/2023	*A. longirostre* 1 M	4838
*C. prehensilis*	Found near the Brasília Zoo	Brasília - DF	17/10/2023	*A. longirostre* 1 M; *A. dubitatum* 2 N	4838
*C. prehensilis*	Found in the Tororó region	Santa Maria - DF	23/11/2023	*A. longirostre* 1 M	4838
*C. prehensilis*	Hotel sector - Taguatinga sul	Brasília - DF	11/10/2024	*A. sculptum* 2 N; *A. longirostre* 1 F	4973
*C. prehensilis*	Farm sector - EPIA DF BR 003 Farm 70, Candangolândia	Brasília - DF	04/12/2024	*A. longirostre* 2 M	4974
**ORDER PRIMATES**					
*Sapajus libidinosus*	BR 251, Chácara Beija Flor KM 49 - São Sebastião	Brasília - DF	16/12/2024	*A. sculptum* 3 N	4972

1 Animals referred by third parties, so it was not possible to determine exactly their places of origin;

+ Contain specimens in which rickettsial DNA was detected (see text);

* All ticks were used for extraction. No ticks from this animal were deposited in the collection;

2 CNC: Coleção Nacional de Carrapatos Danilo Gonçalves Saraiva.

In total, 541 tick specimens were collected from wild animals in this study (3 larvae, 246 nymphs, 163 males, and 129 females). These specimens were identified as belonging to three genera and nine different species. The tick collection results are summarized in Table 3. Tick specimens not submitted for DNA extraction were deposited in the CNC tick collection under the accession numbers CNC-4835, CNC-4836, CNC-4837, CNC-4838, CNC-4839, CNC-4840, CNC-4841, CNC-4842, CNC-4843, CNC-4972, CNC-4973, CNC-4974, CNC-4975, CNC-4976, CNC-4977 and CNC-4978.

**Table 3:** Ticks collected from wild animals necropsied by the Diagnostic Pathology Service at the Veterinary Pathology and Forensic Laboratory, Veterinary Hospital, University of Brasília, during 2022-2024.

**Tick species**	**No. specimens per stage**				**Hosts**
	**Larvae**	**Nymphs**	**Males**	**Females**	**(no. of infested individuals)**
*Amblyomma sculptum*		105	67	78	*Tapirus terrestris* (7)
		1			*Subulo gouazoubira* (1)
		9	46	24	*Hydrochoerus hydrochaeris* (8)
		81		1	*Myrmecophaga tridactyla* (2)
		16			*Cariama cristata* (1)
				1	*Didelphis albiventis* (1)
		6	1		*Coendou prehensilis* (3)
		3			*Aramides cajaneus* (1)
		3			*Sapajus libidinosus* (1)
*Amblyomma longirostre*			15	4	*Coendou prehensilis* (9)
*Amblyomma nodosum*			7	1	*Myrmecophaga tridactyla* (3)
*Amblyomma ovale*				1	*Subulo gouazoubira* (1)
*Amblyomma dubitatum*		1			*Tapirus terrestris* (1)
		17	17	4	*Hydrochoerus hydrochaeris* (8)
					*Coendou prehensilis* (1)
		1			*Aramides cajaneus* (1)
*Amblyomma brasiliense*		1	3	1	*Tapirus terrestris* (2)
*Amblyomma triste*		2	1	1	*Tapirus terrestris* (2)
*Rhipicephalus microplus*	1		5	13	*Subulo gouazoubira* (2)
*Ixodes loricatus*			1		*Didelphis albiventis* (1)
*Amblyomma* sp*.*	2				*Tapirus terrestris* (1)
**Total**	**541 (3 larvae, 246 nymphs, 163 males, 129 females)**				

A total of 15 tick specimens (only intact specimens that had been properly preserved in alcohol were selected; those that appeared to have died before being preserved in ethanol were excluded) were selected for individual DNA extraction: 14 from the genus *Amblyomma* [1 female *Amblyomma ovale* Koch, 1844; 2 males and 1 nymph *Amblyomma dubitatum* Neumann, 1899; 2 males and 1 female *Amblyomma nodosum* Neumann, 1899; and 5 males and 2 females *Amblyomma longirostre* (Koch, 1844)] and 1 specimen from the genus *Ixodes* (1 male *Ixodes loricatus* Neumann, 1899). Following extraction, only one sample - a male *A. nodosum* - was negative in the conventional PCR targeting the mitochondrial 16S rRNA gene present in ectoparasites and was therefore excluded from further PCR assays.

In the PCR assays targeting the genera *Borrelia* and *Coxiella*, as well as the order Piroplasmida, all samples tested negative. In the PCR protocol targeting the family Anaplasmataceae, two specimens of *A. nodosum* (one male and one female) collected from *M. tridactyla* from Brasília (Lago Norte) tested positive. The partial 16S rRNA gene sequences obtained from these samples were identical to each other and 99.7% (305/306 bp) identical to a sequence of ‘*Candidatus* Allocryptoplasma sp.’ from GenBank (OQ724853).

A total of eight tick specimens revealed rickettsial DNA through the TaqMan real-time PCR assay. These ticks were submitted to conventional PCR assays targeting the *gltA* and *ompA* genes, whose products generated DNA sequences that were identified to species based on BLAST analysis. An *A. longirostre* male collected on *C. prehensilis* from Chapada dos Veadeiros (Alto Paraíso, GO) generated *gltA* (324 bp) and *ompA* (444 bp) sequences that were 100% identical to *R. amblyommatis* from GenBank (MZ333467 and MN336348, respectively). Two *A. longirostre* (male and female) collected on *C. prehensilis* from Brasília (CETAS-DF) generated *gltA* (350 bp) and *ompA* (402bp) sequences that were 99.7% and 100%, respectively, identical to *R. amblyommatis* (MG712729 and AY360213, respectively). One *A. dubitatum* nymph collected on *T. terrestris* from Brasília (CETAS-DF) generated *gltA* (350 bp) and *ompA* (590 bp) sequences that were 100% identical to *Rickettsia* sp. strain COOPERI (AY362704 and KM116017, respectively). One *A. ovale* female collected on *S. gouazoubira* from Brasilia (ICMBio) generated *gltA* (350 bp) and *ompA* (434 bp) sequences that were 100% identical to *R. parkeri* strain Atlantic rainforest from GenBank (KJ855083 and JQ906784, respectively).

One *I. loricatus* collected on *D. albiventris* from Brasília (CETAS-DF) generated a *gltA* (229 bp) sequence identical to *R. bellii* (DQ146481), while one *A. longirostre* male collected on *C. prehensilis* from Brasília (CETAS-DF) generated a *gltA* (171 bp) sequence identical to *R. bellii* (KU557517). These two tick specimens, together with an *A. nodosum* male from *M. tridactyla* from Brasília (Lago Norte), yielded amplicons of the expected size through the *R. bellii-*specific PCR assay.

The DNA sequences generated in the present study were deposited in GenBank under the following accession numbers: PX396818 for ‘*Candidatus* Allocryptoplasma sp. 16S rRNA; PX400212-PX400213 for *R. amblyommatis gltA*; PX400218-PX400219 for *R. amblyommatis ompA*; PX400214, PX400220 for *Rickettsia* sp. strain COOPERI *gltA* and *ompA*; PX400215, PX400221 for *R. parkeri* strain Atlantic rainforest *gltA* and *ompA*, PX400216-PX400217 for *R. bellii gltA.*

## Discussion

Through the inspection of the carcasses of 35 necropsied vertebrates at the Diagnostic Pathology Service of a veterinary hospital in central Brazil, it was possible to obtain a wealth of nine species of ticks, comprising three genera of Ixodidae: *Amblyomma, Ixodes,* and *Rhipicephalus*. In addition, it was possible to identify at least three species of bacteria of the genus *Rickettsia* in some of these ticks, as well as a possible novel agent of the Anaplasmataceae family. These results attest to the scientific opportunities that veterinary pathology services can offer for studies on ticks and tick-borne diseases, especially when they include wild animals from their natural habitats.

The tick *Amblyomma sculptum* Berlese, 1888 is one of the most generalist and widely distributed species in Brazil, occurring in all states of the Central-Western region, including the Federal District (Bassini-Silva et al., 2024). The presence of this tick on all hosts in the present study, both birds and mammals, supports the literature data regarding its low host specificity and its great importance as the most frequent and abundant tick species in central Brazil (Martins et al., 2011, 2016).

The tick *A*. *dubitatum* shows a greater preference for parasitizing capybaras (*H. hydrochaeris*), so that its geographic distribution is closely related to the distribution of capybaras in Brazil; i.e., there are no records of this tick in areas without capybaras (Nava et al., 2010). Records of *A. dubitatum* on *H. hydrochaeris* span the three states that comprise the Central-West region, including Goiás (Bastos et al., 2016; Machado et al., 2018; Bassini-Silva et al., 2024), and the Federal District (Quadros et al., 2021), thus corroborating the present findings in Brasília. Besides capybaras, *A. dubitatum* has also been found on other hosts species, such as several species of birds and mammals, including tapirs (*T. terrestris*) in the Brazilian Central-Western region (Nava et al., 2010; Labruna et al., 2021; Luz et al., 2023). Here, we also observed the association between *A. dubitatum* and *T. terrestris*, but during the nymphal stage, representing the first record of this stage on this host. In the present study, *A. dubitatum* nymphs were also found on porcupines (*C. prehensilis*) and on a bird (*Aramides cajaneus*), the latter being the first record of *A. dubitatum* on this avian host species.

The species *Amblyomma triste* Koch, 1844 has previously been recorded parasitizing *T. terrestris* in the Emas National Park, Goiás (Martins et al., 2011; Paludo et al., 2024), aligning with the present identification of *A. triste* on the same host. On the other hand, we report for the first time the species *Amblyomma brasiliense* Aragão, 1908 in the Central-Western region of Brazil, specifically in the Federal District, parasitizing *T. terrestris*. Previous records of *A. brasiliense* have been mostly from the Atlantic forest biome, where this tick is usually associated with forested humid habitats (Szabó et al., 2009; 2013; 2018; Krawczak et al., 2022). This condition might explain the absence of previous records from the Central-Western region of Brazil, largely occupied by the Cerrado biome, in which dense and humid forests are more limited and sparsely distributed (Scariot et al., 2005). Even so, the few records of *A. brasiliense* in the Cerrado biome of southeastern Brazil (Szabó et al., 2018) could corroborate the presence of this tick in some isolated points of this biome in the Central-Western region. Our findings of *A. brasiliense* on two individual tapirs (*T. terrestris*) from different years (2022 and 2024; Table 2), represented by different life stages of the tick (nymph, male and female), strongly indicate that *A. brasiliense* could be established in the Federal District or in its surroundings areas of the Goiás state.

The tick *A. ovale* is widely distributed in the Central-Western region, mainly associated with domestic and wild carnivores (Bassini-Silva et al., 2024). In this study, a female *A. ovale* was found on a brown brocket (*S. gouazoubira*). The species *Rhipicephalus microplus* (Canestrini, 1888) has also been found in the Central-Western region parasitizing several deer species, including *S. gouazoubira* (Witter et al., 2016; Campos Pereira et al., 2000; Prati et al., 2023), corroborating the findings of this study.

In the Central-Western region, *A. longirostre* has been found parasitizing *C. prehensilis* in several municipalities (Labruna et al., 2002; Bastos et al., 2016; Witter et al., 2016), which is consistent with our findings. In the state of Goiás, the association of *A*. *nodosum* with the giant anteater (*Myrmecophaga tridactyla*) has been previously reported in several municipalities (Martins et al., 2011; Machado et al., 2018; Prati et al., 2023), thus supporting the current study. The present finding of *I. loricatus* on the white-eared opossum *Didelphis albiventris* in Brasília is consistent with a previous record in Goiás (municipality of Anápolis), where the same tick-host association was reported (Barros-Battesti & Knysak, 1999).

With regard to the pathogens investigated, two *Rickettsia* species of the spotted fever group, *R. amblyommatis* and *R. parkeri* strain Atlantic rainforest, were detected in *A. longirostre* and *A. ovale,* respectively. While the pathogenicity of *R. amblyommatis* for humans remains under debate (Richardson et al., 2023), *R. parkeri* strain Atlantic rainforest has been confirmed as the agent of several human cases of spotted fever clinical illness in the Atlantic forest biome of Brazil, where *A. ovale* is its main vector (Sevá et al., 2019). Our findings corroborate previous studies that detected this pathogen in *A. ovale* from the Central-western region of Brazil (Prati et al., 2023), where human cases may be occurring but are confused with other clinically similar diseases, such as dengue and other arboviruses.

Our finding of *Rickettsia* sp. strain COOPERI in an *A. dubitatum* nymph corroborates previous studies that reported this agent in *A. dubitatum* ticks from Brasilia (Quadros et al., 2021) and São Paulo (Labruna et al., 2004). This agent belongs to the spotted fever group, and it could represent a new strain of *R. parkeri* potentially pathogenic for humans (Nieri-Bastos et al., 2018). Finally, our findings of *R. bellii* on three different tick species (*A. longirostre, A. nodosum* and *I. loricatus*) is in accordance with previous studies that reported this non-pathogenic agent in various tick species of the New World (Krawczak et al., 2018).

The bacterial Anaplasmataceae family contains tick-borne pathogenic organisms of the genera *Anaplasma* and *Ehrlichia* (Hördt et al., 2020; Oren & Garrity, 2022). While none of these agents were detected in this study, we surprisingly amplified from two *A. nodosum* ticks a 16S rRNA fragment that matched 99.7% to a sequence of ‘*Candidatus* Allocryptoplasma sp.’, previously detected in *Amblyomma tholloni* (Neumann, 1906) from Uganda (Ouass et al., 2023). Different haplotypes of ‘*Candidatus* Allocryptoplasma sp.’ have been detected in hard ticks, mammals, birds and reptiles (Ouass et al., 2023, Córdova et al., 2024), suggesting that this putative bacterial genus of the Anaplasmataceae family could represent agents of tick-borne infections, yet to be confirmed (Ouass et al., 2023). To our knowledge, the only previous report of ‘*Candidatus* Allocryptoplasma sp.’ in ticks from Brazil was in *Amblyomma dissimile* Koch, 1844 from Pará state, Amazon region (Ogrzewalska et al., 2019).

All tick samples processed in this study tested negative for Piroplasmida and bacteria of the genera *Borrelia* and *Coxiella*. It is worth noting that two species of the genus *Amblyomma* from Argentina, *Amblyomma tigrinum* Koch, 1844 and *Amblyomma parvum* Aragão, 1908 (which also occur in Central-Western Brazil), were found naturally infected by *C. burnetii* (Pacheco et al., 2013). On the other hand, *Coxiella-*like endosymbionts (CLEs) have been reported in several South American *Amblyomma* species (Machado-Ferreira et al., 2016), including *A. sculptum* from Brazil (Machado-Ferreira et al., 2011). The lack of knowledge regarding the pathogenicity of CLEs, competence and vector capacity of most naturally infected ticks (Duron, 2015), as well as the role of wild animals in the enzootic cycle of *C. burnetii*, highlights the importance of investigating this pathogen in ticks collected from wild animals.

## Conclusion

A richness of nine species of hard ticks was found on wild animals from a veterinary pathology service in central Brazil, including the first reports of *A. dubitatum* nymphs on the bird *A. cajaneus* and on the tapir *T. terrestris.* In addition, the tick species *A. brasiliense* is reported for the first time in the Central-Western region of Brazil. Finally, four rickettsial agents were reported in ticks, notably *R. parkeri* strain Atlantic rainforest in *A. ovale*, since this *Rickettsia* has been implicated as the agent of clinical cases of spotted fever in Brazil. This study represents a significant contribution to the understanding of the geographic distribution and host associations between ticks and wild animals. The proximity between humans and wild animals may facilitate the transmission of agents of tick-borne zoonoses, especially among professionals working in veterinary pathology laboratories involved in anatomical and pathological diagnostics.

## Data Availability

Data availability will be made available on request.
